# Risk factors of acute ischemic stroke in patients presented to Beni-Suef University Hospital: prevalence and relation to stroke severity at presentation

**DOI:** 10.1186/s41983-018-0012-4

**Published:** 2018-04-25

**Authors:** Rasha H. Soliman, Mohammed I. Oraby, Mohammed Fathy, Alaa M. Essam

**Affiliations:** 1 Department of Neurology, Beni-Suef University, Beni-Suef, 62511 Egypt; 2 Department of Cardiology, Beni-Suef University, Beni-Suef, Egypt

**Keywords:** Acute ischemic stroke, Risk factors, Beni-Suef, Stroke severity

## Abstract

**Background:**

Acute ischemic stroke is one of the major causes of disability and death worldwide. Effective prevention remains the best approach for reducing the burden of stroke. The aim of this work was to study the prevalence of stroke risk factors and the possible relation between such risk factors and the disease severity at presentation in a sample of stroke patients presented to Beni-Suef University Hospital, north Upper Egypt.

**Methods:**

A sample of 167 patients of acute ischemic stroke recruited from Beni-Suef University Hospital was included in this cross-sectional descriptive study. All subjects were subjected to history taking, clinical, laboratory, and radiological evaluation. Stroke severity and disability were evaluated by National Institute of Health Stroke Scale (NIHSS) and the modified Rankin Scale (mRS) respectively.

**Results:**

Hypertension was detected in 104 patients (62.3%), dyslipidemia was detected in 79 patients (58.1%), and 69 patients (41.3%) were smokers. Diabetes mellitus was detected in 58 patients (34.7%) with high prevalence of cardio-embolic risk factor, 36 patients (21.6%) had rheumatic heart, and 44 patients (26.3%) had atrial fibrillation.

NIHSS score was significantly higher in hypertensive patients (*P* value = 0.023) and in patients who had carotid stenosis ≥ 50% (*P* value = 0.011), whereas there was no significant relation between NIHSS score and diabetes mellitus (*P* = 0.221), dyslipidemia (*P* = 0.834), patients with history of cardio-embolic stroke (*P* = 0.085), previous ischemic stroke (*P* = 0.316), or sex (*P* = 0.343).

mRS score was significantly higher in patients with age > 45 years old (*P* < 0.001), hypertension (*P* < 0.001), cardio-embolic risk factor (*P* = 0.044), and carotid stenosis ≥ 50% (*P* = 0.017), whereas there was no significant relation between mRS score and diabetes mellitus, previous ischemic stroke, or sex.

**Conclusions:**

The most common risk factor for stroke was hypertension followed by dyslipidemia and then smoking with higher incidence of rheumatic heart diseases due to lowered living conditions. Age, hypertension, cardio-embolic risk factors, and carotid stenosis ≥ 50% have negative impact on stroke severity and disability.

## Background

Stroke is a catastrophic life-changing event which influences not only stroke patients but also their families and care givers (Lloyd-Jones et al. 2009).

Progress has been made in reducing deaths from stroke. Despite the advent of intravenous tissue-type plasminogen activator as a specific treatment for selected patients with acute ischemic stroke in therapeutic window and the promise of other intervention therapies, still the best approach to decrease stroke burden is effective prevention (Adams et al. 2007).

Stroke is considered as a disease which can be developed by long-lasting exposure to risk factors related to lifestyle. Modification of such risk factors should greatly affect the incidence of stroke and even mortality rates (Scarborough et al. 2011). Primary prevention is particularly important because 77% of strokes are first events. The risk of a first stroke can be lowered by 80% in people who practice a healthy lifestyle compared with those who do not (Chiuve et al. 2008).

Different modifiable and non-modifiable risk factors have been recognized for stroke. Non-modifiable risk factors are gender, age, ethnicity, heredity, and race. Modifiable risk factors include, but are not limited to, hypertension, dyslipidemia, diabetes mellitus, atrial fibrillation, smoking, drug abuse, and alcoholic intake (Zafar et al. 2016).

Beni-Suef is one of the Upper Egypt governorates located about 120 km south of Cairo mostly in the west bank of the Nile River. According to population estimates from 2015, the majority of residents in the governorate live in rural areas, with an urbanization rate of only 23.2% (CAPMAS 2014).

## Methods

The aim of this work was to study the prevalence of stroke risk factors and the possible relation between such risk factors and the disease severity at presentation in a sample of stroke patients presented to Beni-Suef University Hospital, north Upper Egypt.

This study is a cross-sectional descriptive study and was conducted on a sample of 167 patients of acute ischemic stroke presented within 24 h from onset, 15–90 years old, attending the outpatient clinic and emergency room in Beni-Suef University Hospital, from June 2015 to August 2016.

Patients with hemorrhagic stroke and patients with venous infarction were excluded from the study. A written informed consent was obtained from each participant in this study or from one of his family members, and the study was approved by the local ethical committee in Faculty of Medicine, Beni-Suef University.

All patients included in this study were subjected to the following:

1. Detailed history taking with special focusing on the following items:
  ▪ Hypertension: patient’s self-report of hypertension or use of antihypertensive drugs.
  ▪ Diabetes: patient’s self-report of diabetes or use of antidiabetic drugs.
  ▪ Smoking: former smokers (adults who have smoked at least 100 cigarettes in their lifetime, stopped smoking recently) or current smokers (adults who have smoked 100 cigarettes in their lifetime and currently smoke cigarettes daily or some days (nondaily) (US Centers for Disease Control and Prevention 2010).
  ▪ Alcohol abuse: alcohol consumption was regarded chronic heavy drinking when a patient regularly takes > 3 heavy drinks per day (> 36 g of alcohol/day) and as an acute alcohol intoxication when a patient had taken > 48 g of alcohol during preceding 24 h (Mazzaglia et al. 2001).
  ▪ Family history [paternal and maternal] of stroke.
  ▪ Previous history of stroke or transient ischemic attacks.
  ▪ Oral contraceptives: oral contraceptives were considered risk factor if they were used at any time in a 3-month period before the stroke (World Health Organization Collaborative Study of Cardiovascular Disease and Steroid Hormone Contraception 1996).
  ▪ Stresses: hours before stroke.
2. Through general and neurological examination including detailed cardiological assessment and neurovascular examination according to the cerebrovascular stroke assessment sheet of Neurology Department, Beni-Suef University, body mass index (BMI) is calculated as weight in kilograms divided by height in meters squared. The following ranges are generally used to determine risks related to weight: underweight < 18.5, normal 18.5–24.9, overweight 25.0–29.9, and obese ≥ 30 and morbidly obese ≥ 40 (National Institutes of Health (NIH), National Heart, Lung, and Blood Institute (NHLBI) 2000).
3. Stroke severity assessed by the National Institute of Health Stroke Scale (NIHSS) (NIH Stroke Scale) and the modified Rankin Scale (mRS) (Bonita and Beaglehole 1988).
4. Routine laboratory investigations including:
  ▪ Complete blood count, liver functions, prothrombin time and concentration and international normalized ratio (INR), kidney functions, fasting blood sugar, 2 h post prandial, and glycated hemoglobin (HbA1C). Diabetes is diagnosed by demonstrating any one of the following: fasting plasma glucose level ≥ 126 mg/dl, plasma glucose ≥ 200 mg/dl 2 h after a 75-g oral glucose load as in a glucose tolerance test, or glycated hemoglobin (HbA1C) ≥ 6.5% (WHO 2006).
  ▪ Lipid profile: total cholesterol, low-density cholesterol, and high-density cholesterol. According to Adult Treatment Panel (ATP) III, total cholesterol ≥ 240 mg/dl, LDL ≥ 160 mg/dl, and HDL < 40 mg/dl are considered at high risk of atherosclerosis and coronary heart disease (Expert Panel on Detection, Evaluation, and Treatment of High Blood Cholesterol in Adults 2001).
5. Radiological assessment:
  ▪ Brain computed tomography (CT): using Toshiba Multislice 16 computed tomography in radiology department in Beni-Suef University Hospital.
  ▪ Electrocardiography (ECG): using standard 12 leads electrocardiogram.
  ▪ Echocardiography: vivid S5 machine transthoracic echo-Doppler study using 2D and M mode was done by trained cardiologist in cardiology department in Beni-Suef University Hospital.
  ▪ Carotid duplex: Toshiba machine carotid duplex using B mode and Doppler flow was done by radiologist in radiology department in Beni-Suef University Hospital.
  In selected cases, in young patients (15–45 years old), if no obvious risk factor was found, the following investigations were done:

- ▪ Transesophageal echocardiography (TEE): by vivid S5 machine TEE was done using 2D, colored Doppler, and agitated saline in cardiology department in Beni-Suef University Hospital.
- ▪ Immunological tests: antinuclear antibody, anti-neutrophil cytoplasmic antibody, anti-double strand DNA, rheumatoid factor, anticardiolipin antibody, and lupus anticoagulant.
- ▪ Proteins C and S and antithrombin III.
- ▪ Detection of factor V Leiden mutation.

### Statistical analysis

Data were statistically described in terms of range, mean ± standard deviation (± SD), median, frequencies (number of cases), and relative frequencies (percentages) when appropriate. Comparison of quantitative variables between the study groups was done using independent samples *t* test. For comparing categorical data, chi-squared (*χ*^2^) test was performed. A probability value (*P* value) less than 0.05 was considered statistically significant. All statistical calculations were done using computer programs: Microsoft Excel version 7 (Microsoft Corporation, NY, USA) and SPSS version 20 (Statistical Package for the Social Science; SPSS Inc., Chicago, IL, USA) statistical program for Microsoft Windows.

## Results

One hundred sixty-seven patients with acute ischemic stroke were included in this cross-sectional descriptive study. Their age ranged from 15 to 90 years, with mean and standard deviation of 59.3 ± 13.45 years. In 90 males (53.9%), 11 patients of them were ≤ 45 years old, and in 77 females (46.1%), 13 patients of them were ≤ 45 years old. The frequency of risk factors in acute ischemic stroke patients is shown in Table 1.

**Table 1:** Distribution of risk factors in 167 studied ischemic stroke patients

Risk factors	Number	Percent
Hypertension	104	62.3
Diabetes	58	34.7
Smoking	69	41.3
Alcohol intake	0	0
Positive family history	23	13.8
History of TIA	9	5.4
History of previous stroke	46	27.5
Stress	9	5.4
Obesity	20	12
Cardio-embolic	60	35.9
Significant carotid stenosis (≥ 50%)	6	3.6
Dyslipidemia	97	58.1
Migraine	4	2.4
Pregnancy and postpartum	3	3.9
Oral contraceptive	2	12.5

### Clinical evaluation and investigations

Assessment of stroke severity and disability at presentation were carried out using National Institute of Health Stroke Scale (NIHSS) and modified Rankin Scale (mRS) respectively (Table 2). The mean of NIHSS score was 10.25 ± 5.83 (range 1–27) and for mRS was 3.57 ± 1.03 (range 1–5).

**Table 2:** Results of NIHSS and mRS in 167 ischemic stroke patients

Scale	Score	Number (167)	Percent
NIHSS	Mild (1–4)	33	19.8
	Moderate (5–15)	103	61.7
	Moderate to severe (16–20)	19	11.4
	Severe (21–42)	12	7.2
mRS	0	0	0
	1	6	3.6
	2	22	13.2
	3	43	25.7
	5	29	17.4
	6	0	0

Cardiac evaluation in 167 patients of ischemic stroke is shown in Table 3.

**Table 3:** Results of cardiological assessment in 167 ischemic stroke patients

Cardiac risk factors	Number (167)	Percent
Rheumatic heart disease	36	21.6
Atrial fibrillation	44	26.3
Atrial myxoma	1	0.6
Prosthetic valve	3	1.8
Mural thrombus	3	1.8
Ischemic heart disease	15	9
Left ventricular hypertrophy	51	30.5

Carotid duplex using B mode and Doppler flow revealed that 94 patients had increased intimal medial thickness and 28 patients (16.8%) had carotid artery stenosis; 22 patients of them had non-significant stenosis (less than 50%), 4 patients had significant carotid artery stenosis (more than 50% stenosis), and 2 patients had totally occluded contralateral carotid vessel with non-significant stenosis of the ipsilateral carotid vessel.

Twelve patients with stroke in young age (≤ 45 years) who have no obvious cardio-embolic risk factors were selected for specific tests: transesophageal echocardiography (TEE), immunological tests (ANA, ANCA, anti-double strand DNA, anticardiolipin, and lupus anticoagulant), and thrombophilic profile (proteins C and S, antithrombin III, and factor V Leiden) (Table 4).

**Table 4:** Distribution of additional risk factors in 12 ischemic stroke patients ≤ 45 years old

Risk factors	Total number = 12	
	Number	Percent
Auto-immune vasculitis	0	0
Antiphospholipid syndrome	1	8.3
Protein C deficiency	2	16.7
Protein S deficiency	1	8.3
Antithrombin III deficiency	0	0
Factor V Leiden mutation	9	75.0
Patent foramen ovale	1	8.3
Undetermined	1	8.3

### Relation of risk factors to stroke severity and disability at presentation using NIHSS and mRS

Table 5 shows the relation between non-modifiable risk factors and stroke severity and disability at presentation using NIHSS and mRS. The only statistically significant difference was between age and stroke disability at presentation using mRS, disability was higher in patient with age > 45 years (*P* value < 0.001).

**Table 5:** Relation between non-modifiable risk factors and stroke severity and disability at presentation using NIHSS and mRS

Risk factors	NIHSS		mRS	
	Mean ± standard deviation	*P* value	Mean ± standard deviation	*P* value
Age				
15–45	8.25 ± 4.84	0.092	2.75 ± 0.99	< 0.001*
46–90	10.58 ± 5.92		3.71 ± 0.97	
Sex				
Male	10.01 ± 6.29	0.343	3.49 ± 1.06	0.359
Female	10.52 ± 5.26		3.66 ± 0.98	
Family history				
+ve	10.09 ± 5.45	0.909	3.35 ± 1.15	0.296
−ve	10.27 ± 5.90		3.60 ± 1.01	
History of TIA				
Yes	10.33 ± 4.39	0.725	4.00 ± 0.71	0.215
No	10.24 ± 5.91		3.54 ± 1.04	
History of previous stroke				
Yes	10.67 ± 5.19	0.316	3.83 ± 0.80	0.063
No	10.08 ± 6.06		3.47 ± 1.09	
Pregnancy and postpartum				
Yes	6.33 ± 5.77	0.364	2.67 ± 1.15	0.439
No	9.00 ± 4.71		3.15 ± 0.99	

*P is significant if < 0.05

The relation of modifiable risk factors to severity of ischemic stroke at presentation using NIHSS score is shown in Table 6. NIHSS score was significantly higher in hypertensive patients (*P* value = 0.023) and in patients who had carotid stenosis ≥ 50% (*P* value = 0.011). There was no significant relation between NIHSS score and diabetes mellitus (*P* = 0.221), dyslipidemia (*P* = 0.834), or patients with history of cardio-embolic stroke (*P* = 0.085).

**Table 6:** Relation between modifiable risk factors and ischemic stroke severity and disability at presentation using NIHSS and mRS

Risk factor	NIHSS		mRS	
	Mean ± standard deviation	*P* value	Mean ± standard deviation	*P* value
Hypertension				
Yes	11.05 ± 5.91	0.023*	3.80 ± 0.94	< 0.001*
No	8.92 ± 5.48		3.19 ± 1.06	
Diabetes mellitus				
Yes	9.43 ± 5.18	0.221	3.60 ± 0.97	0.927
No	10.68 ± 6.12		3.55 ± 1.06	
Smoking				
Yes	10.29 ± 6.37	0.766	3.57 ± 1.01	0.901
No	10.21 ± 5.45		3.57 ± 1.05	
Obesity				
Yes	10.15 ± 6.97	0.634	3.30 ± 1.17	0.211
No	10.26 ± 5.68		3.61 ± 1	
Cardio-embolic				
Yes	11.52 ± 6.64	0.085	3.75 ± 1.10	0.044*
No	9.53 ± 5.21		3.47 ± 0.97	
Carotid stenosis				
≥ 50%	17.17 ± 6.31	0.011*	4.50 ± .55	0.017*
< 50%	9.99 ± 5.67		3.53 ± 1.2	
Dyslipidemia				
Yes	10.33 ± 5.88	0.834	3.28 ± 1.16	0.201
No	10.13 ± 5.79		3.58 ± 1.01	

*P is significant if < 0.05

The relation of modifiable risk factors to ischemic stroke disability at presentation using mRS is shown in Table 6. mRs was significantly higher in hypertensive patients compared to non-hypertensive patients (*P* value < 0.001), patients with cardio-embolic risk factor compared to patients without cardio-embolic risk factor (*P* value = 0.044), and between patients with carotid stenosis ≥ 50% compared to patients with carotid stenosis < 50% (*P* value = 0.017).

Table 7 shows the relation of potentially modifiable risk factors to severity and disability of ischemic stroke at presentation using NIHSS and mRS respectively and revealed that there was no statistically significant relation between any of such risk factors and NIHSS or mRS.

**Table 7:** Relation between potentially modifiable risk factors and ischemic stroke severity and disability at presentation using NIHSS and mRS

Risk factor	NIHSS		mRS	
	Mean ± standard deviation	*P* value	Mean ± standard deviation	*P* value
Migraine				
Yes	7.50 ± 6.61	0.266	3.00 ± 1.41	0.265
No	10.31 ± 5.81		3.58 ± 1.02	
Antiphospholipid syndrome				
Yes	16.00	0.333	3.00	0.833
No	7.82 ± 4.87		2.55 ± 0.93	
Factor V Leiden mutation				
Yes	8.00 ± 5.41	0.600	2.44 ± 1.01	0.482
No	10.00 ± 5.29		3.00 ± 0.00	
Protein C				
Normal	9.60 ± 5.02	0.121	2.80 ± 0.79	0.121
Deficient	3.00		1.50 ± 0.71	
Protein S				
Normal	9.00 ± 5.16	0.333	2.64 ± 0.92	0.500
Deficient	3.00		2.00	

## Discussion

In developed world, the number of strokes decreased by approximately 10% and, on the other hand, increased by 10% in developing countries between 1990 and 2010 (Feigin et al. 2014). In the Middle East region, stroke is considered as a major health problem which leads to significant disability and mortality with an expected increase in mortality rate which may reach the double by 2030 (Tran et al. 2010).

The most important predictors of outcome in the acute phase of stroke are stroke severity and the age of patient. Stroke severity can be assessed clinically, based on various parameters of neurologic impairment (e.g., altered mentation, motor deficit, language, visual field deficit, behavior) and the size and location of the infarction on neuroimaging with MRI or CT. Ischemic stroke mechanism, epidemiologic factors, comorbid conditions, and complications of stroke are other important factors that have an influence on stroke outcome (König et al. 2008).

The aim of this work is to study the pattern of distribution of stroke risk factors in a sample of stroke patients in Beni-Suef governorate and the possible relation between such risk factors and the disease severity at presentation.

The current study was conducted on 167 patients presented with acute ischemic stroke. Male patients in this study constituted 53.9% while female patients constituted 46.1%; this is in accordance with the study of Grau et al.’s (2001) in which male patients constituted 57.6% and female patients constituted 42.4% and in Altafi et al.’s (2013) study 54.54% were men and 46% were women. Male predominance was also clear in a large prospective multicenter hospital-based stroke registry in Saudi Arabia including only ischemic stroke (Deleu et al. 2011).

The higher prevalence of stroke among males can be explained by the hormonal constitutional factors plus the higher rate of smoking and higher rate of stressful situations among males than females (most females in this study are housewives) (El Tallawy et al. 2015).

In the present study, incidence of cerebral infarction increased with advancing age where 85.6% of the patients were between 46 and 90 years and 14.4% of the patients were ≤ 45 years.

The current study is in agreement to the previous studies showing that incidence of cerebral infarction increased with advancing age in Marwat et al.’s (2009) study, incidence of cerebral infarction increased with advancing age where 2.3% in the age group 40–50, 27.2% in the age group 51–60, and 47.7% in the age group older than 60 years. Also, in Grau et al.’s (2001) study, ischemic stroke increased with advancing age where 5.7% of the patients were < 45 years and 94.3% of the patients were ≥ 45 years.

Aging has cumulative effects on the cardiovascular system, and the progressive nature of stroke risk factors over a prolonged period ultimately increases the risks of both ischemic stroke and intracerebral hemorrhage (Carandang et al. 2006).

Evaluation of current and other studies showed that hypertension is one of the most prevalent cardiovascular risk factors; this is probably due to the high prevalence of this disease in older patients. In the present study, hypertension was the most common risk factor for ischemic stroke, which was detected in 62.3% of all studied cases.

This is in accordance with many old and recent studies (Grau et al. 2001; Hatano 1973). Hypertension has also been described as the most commonly identified risk factor for both ischemic and hemorrhagic strokes in studies from Egypt and other Arab countries (El Tallawy et al. 2015; Essa et al. 2011; El Sayed et al. 1999).

A linear relationship between blood pressure and stroke risk has been consistently declared. In prospective studies, every 10 mmHg reduction in BP was associated with a 33% lowering of stroke risk in primary prevention (Lawes et al. 2004).

Different mechanisms can explain the negative impact of hypertension in a cerebrovascular autoregulation which likely include a combination of the changes on the mechanical characteristics of cerebral blood vessels induced by remodeling and stiffening and effects on myogenic tone. These changes in autoregulation are particularly damaging the periventricular white matter, which is located at the boundary zone between different arterial territories and more liable to hypoperfusion (Shinton and Beevers 1989).

In our study, diabetes mellitus was recorded in 34.7% of patients which is slightly lower than El Tallawy et al.’s (2015) study done in Upper Egypt where diabetes mellitus was recorded in 36.5% of patients, whereas in Essa et al.’s (2011) study in Alexandria, diabetes mellitus was recorded in 66.8% of patients.

Diabetes is associated with increased level of coagulation factors and hyperinsulinemia that have an important role in the development of microangiopathic stroke. Also, macroangiopathic infarction can occur as diabetes accelerates the atherosclerotic process of large cerebral arteries (Tanizaki et al. 2000).

In the current study, dyslipidemia was recorded in 58.1% of all patients which is higher than Grau et al.’s (2001) study where dyslipidemia was recorded in 35.3% of patients and slightly higher than what was recorded by El Tallawy et al.’s (2015) study done in Upper Egypt, where dyslipidemia was recorded in 54.2% of patients.

The potential role of lipids as stroke risk factor is less clear compared to coronary or peripheral vascular disease; this is mostly attributed to the heterogeneity of stroke (e.g., hemorrhagic stroke, non-atherosclerotic subtypes of ischemic stroke) (Go et al. 2013). Despite this, the risk of stroke can be reduced in a number of patient populations including those with coronary heart disease, diabetes, hypertension, and the elderly by receiving HMG-CoA reductase inhibitors (statins) (Amarenco et al. 2004). Every 1-mmol decrease in LDL-C was associated with a 17% reduction in fatal and nonfatal stroke (Baigent et al. 2005).

Atrial fibrillation was associated with 26.3% of ischemic stroke in our study which is higher than 17.2% of ischemic stroke found in the study of Saposnik et al. (2013).

This can be explained by higher rheumatic heart disease incidence in our community, which was recorded in 21.6% of our patients where 77.8% of them had AF. This is attributed to low living conditions, which have resulted in more overcrowding and worse hygiene, with consequent elevation in transmission of group A streptococci.

Positive parental family history was recorded in 13.8% of our ischemic stroke patients. A meta-analysis of cohort studies showed that a positive family history of stroke increases risk of stroke by approximately 30% (Flossmann et al. 2004).

Different mechanisms can explain the possible role of a positive family history on increasing the risk of stroke, including genetic heritability of stroke risk factors, inheritance of susceptibility to the effects of such risk factors, familial sharing of cultural/environmental and life style factors, and interaction between genetic and environmental factors (Flossmann et al. 2004).

History of previous stroke was recorded in 27.5% of patients included in this study which is higher than results from a previous study done by Altafi et al.’s study (2013) who found that history of previous stroke was associated with 26% of ischemic stroke cases.

Most of our patients with history of previous stroke were not compliant to their medications and with no proper control of their modifiable risk factors including blood pressure, diabetes mellitus, dyslipidemia, and target INR in cardio-embolic strokes. This can explain the high incidence of recurrence of stroke in our studied population.

Cigarette smoking is a well-known stroke risk factor and has a strong association with athero-thrombotic process. In the Framingham study, it was found that the relative risk of stroke in smokers, after adjusting for age and hypertension, was 2.3 in men and 3.1 in women. Also, a significant dose-response relationship was found. In heavy smokers, the risk of stroke was doubled compared with light smokers. The risk of stroke returned to nonsmoker levels after 5 years of smoking cessation (Wolf et al. 1988).

In the present study, smoking was recorded in 41.3% which is less than results from an older study done by Shinton and Beevers (1989) who found that smoking was associated with 55% of ischemic stroke cases.

In the present study, factor V Leiden mutation, protein C deficiency, protein S deficiency, and antiphosphlipid syndrome were present in 75, 16.7, 8.3, and 16.7% of young stroke patients (≤ 45 years old) respectively.

Although Hamedani et al. (2010) reported that factor V Leiden was associated with a twofold increased risk of arterial ischemic stroke in patients below 50 years of age, Morris et al. (2010), in their analysis of the case-control study of the five most commonly inherited thrombophilias with ischemic stroke: protein C and S deficiencies, antithrombin deficiency, factor V Leiden, and prothrombin gene mutations, found no convincing associations with stroke, even in young patients and patients with patent foramen oval.

To assess the relation between the previous risk factors and stroke severity at presentation, the relationship between NIHSS level and hypertension, dyslipidemia, diabetes mellitus, cardio-embolic strokes, and history of previous stroke was evaluated.

In the current study, *T* test’s results showed that NIHSS score was significantly higher in hypertensive patients (*P* value = 0.023) and in patients who had carotid stenosis ≥ 50% (*P* value = 0.011), whereas there was no significant relation between NIHSS score and diabetes mellitus (*P* = 0.221), dyslipidemia (*P* = 0.834), patients with history of cardio-embolic stroke (*P* = 0.085), previous ischemic stroke (*P* = 0.316), or sex (*P* = 0.343).

In Altafi et al.’s study (2013), there was no significant relation between NIHSS score with risk factors. While in Kwangsoo’s study (2012), among stroke risk factors, atrial fibrillation was significantly correlated with NIHSS (*P* < 0.001).

The significant negative impact of hypertension and carotid stenosis on NIHSS may be explained by high prevalence of hypertension in older patients; also, age has by far the strongest independent association with carotid atherosclerosis where mean age of patients with significant carotid artery stenosis was 74 years (Fabris et al. 1994). Advancing age has a major negative impact on stroke morbidity (Knoflach et al. 2012).

In our study, the NIHSS was evaluated in smoking and non-smoking patients and no significant difference was found (*P* = 0.901).

Hage (2011) found that smoker patients had lower NIHSS and lower in-hospital mortality after stroke compared to nonsmokers. This can be explained by the ischemic preconditioning in smokers secondary to episodic hypoxia and chronic changes in vasomotor tone and presence of small vessel cerebral collaterals leading to better cerebral perfusion. Nevertheless, this should not be considered as a benefit of cigarette smoking.

Relationship between the degree of disability using mRS and stroke risk factors was evaluated. In the current study, mRS score was significantly higher in patients with age> 45 years old (*P* < 0.001), hypertension (*P* < 0.001), cardio-embolic risk factor (*P* = 0.044), and carotid stenosis ≥ 50% (*P* = 0.017), whereas there was no significant relation between mRS score and diabetes mellitus, previous ischemic stroke, or sex.

In Kwangsoo’s study (2012), among stroke risk factors, atrial fibrillation, hypertension, and age were significantly correlated with mRS (*P* = 0.006, *P* = 0.010, *P* = 0.018, respectively).

The influence of age in stroke outcome was discussed before; patients with strokes of cardio-embolic or large artery etiology tend to have worse prognosis for recovery compared with other ischemic stroke subtypes (Lima et al. 2014). The significant relation between significant carotid artery stenosis and patient disability in our study may be attributed to older age of the patients and the small sample size.

## Conclusions

Finally, we conclude that hypertension was one of the most prevalent risk factor that is probably due to the high prevalence of this disease in older patients. There was a high prevalence of cardio-embolic risk factor due to higher rheumatic heart diseases in our community which is attributed to low living conditions which make patients more susceptible to have AF and subsequently more incidence of cardio-embolic strokes.

The limitation of this study is the relatively small number of patients included, and it is a hospital-based single center study, a larger multicenter study covering the whole governorate or even a community-based study is recommended.

We also recommend multiple awareness campaigns to be done in Beni-Suef to improve community awareness about stroke and its risk factors and a national project to be planned by ministry of health to control important risk factors like hypertension, heart diseases, and diabetes.

## Abbreviations

ANA: Antinuclear antibodies
ANCA: Antineutrophil cytoplasmic antibodies
ATP: Adult Treatment Panel
BMI: Body mass index
CT: Computed tomography
ECG: Electrocardiography
HbA1C: Glycated hemoglobin
HDL: High-density lipoprotein
HMG-CoA: 3-Hydroxy-3-methylglutaryl-coenzyme A
INR: International normalized ratio
LDL: Low-density lipoprotein
mRS: Modified Rankin Scale
NIHSS: National Institute of Health Stroke Scale
TEE: Transesophageal echocardiography
